# Laparoscopic versus open distal gastrectomy for advanced gastric cancer in elderly patients: a propensity-score matched analysis

**DOI:** 10.1186/s12957-023-03269-2

**Published:** 2024-01-09

**Authors:** Qing Yao, Qian-Nan Sun, Dao-Rong Wang

**Affiliations:** 1 Northern Jiangsu People’s Hospital Affiliated to Dalian Medical University, Yangzhou, 225001 China; 2 Northern Jiangsu People’s Hospital, No.98 Nantong West Road, Yangzhou, Yangzhou, 225001 China; 3 General Surgery Institute of Yangzhou, Yangzhou University, Yangzhou, 225001 China; 4 Yangzhou, Key Laboratory of Basic and Clinical Transformation of Digestive and Metabolic, Yangzhou, 225001 China; 5 Medical Research Center of Northern Jiangsu People’s Hospital, Yangzhou, 225001 China

**Keywords:** Elderly patients, Advanced gastric cancer, Laparoscopic, D2 lymph node dissection

## Abstract

**Background:**

Scarce research has reported the efficacy and safety of laparoscopic distal gastrectomy in elderly patients. This retrospective study aimed to compare the outcomes of laparoscopic and open distal gastrectomy for advanced gastric cancer in elderly patients.

**Methods:**

A total of 303 elderly patients who underwent distal gastrectomy for advanced gastric cancer from June 2017 to June 2021 were enrolled. Variables used to calculate propensity score matching included sex, age, body mass index, American Society of Anesthesiologists, history of diabetes, and history of hypertension. The statistical significance of continuous variables was tested using an independent sample *t* test. chi-square or Fisher’s exact tests were used for categorical variables. Kaplan–Meier curve and log-rank test were used for the evaluation of 3-year overall survival and recurrence-free survival.

**Results:**

After performing 1:1 propensity score matching, 248 patients were included for analysis (laparoscopic = 124, open = 124). Compared with the open group, the laparoscopic group showed significant advantages in estimated blood loss (*P* < 0.001), pain scale on the first postoperative day (*P* = 0.002), time to first flatus (*P* = 0.004), time to first liquid diet (*P* = 0.005), hospital stays (*P* < 0.001), and total complications (*P* = 0.011), but devoted much more operation time (*P* < 0.001). No statistical difference was observed between the two groups in 3-year recurrence-free survival (*P* = 0.315) or overall survival (*P* = 0.159).

**Conclusions:**

Our analysis demonstrated that laparoscopic surgery had the advantages of less intraoperative blood loss, fewer postoperative complications, and faster postoperative recovery in distal gastrectomy for advanced gastric, indicating that laparoscopic distal gastrectomy is safe and effective for treating elderly patients with distal gastric cancer.

## Background

Gastric cancer ranks fifth and fourth in global incidence and mortality, causing more than one million new cases and an estimated 769,000 deaths in 2020 [1]. Since Kitano et al. [2] reported the first laparoscopic-assisted distal gastrectomy in 1994, laparoscopy has developed rapidly in gastric cancer surgery. Goh et al. [3] reported a satisfactory short-term outcome after laparoscopic radical gastrectomy with D2 lymph node dissection in patients with advanced gastric cancer. Compared with open surgery, multiple randomized controlled trials (RCT) and large-scale cohort studies have confirmed that laparoscopic distal gastrectomy in advanced gastric cancer has obvious advantages, including intraoperative blood loss, postoperative complications, and postoperative recovery rate [4–7].

The aging population has largely contributed to the increase in new cancer cases worldwide [8, 9]. Among gastric cancer patients in South Korea, the proportion of patients older than 71 years of age increased from 9.1% in 1995 to 28.8% in 2019 [10]. Moreover, the incidence of gastric cancer in elderly patients in Japan is increasing yearly, and more than 30% of gastric cancer patients are over 80 years old [11]. With the development of minimally invasive surgical techniques, laparoscopic surgery has made great progress in the treatment of gastric cancer, but it still faces great challenges for elderly patients [12]. Elderly patients with gastric cancer are more prone to postoperative infectious complications such as pneumonia due to the greater burden of comorbidities and lower functional reserve capacity [13, 14]. Yen et al. [15] conducted a retrospective analysis on 76 gastric cancer patients over 70 years old and found that the laparoscopic distal gastrectomy group had the advantages of shorter hospital stays and fewer surgical complications compared to open surgery.

However, the safety and efficacy of laparoscopic distal gastric cancer surgery in elderly patients are still insufficient. Therefore, the purpose of this study was to perform a retrospective analysis of elderly patients (older than 70 years) undergoing distal gastric cancer surgery in our hospital. We aimed to investigate the intraoperative conditions, postoperative recovery, postoperative complications, and postoperative survival of laparoscopic surgery in elderly patients with gastric cancer.

## Methods

### Patient selection

This retrospective cohort study collected elderly patients who underwent laparoscopic or open distal gastrectomy for advanced gastric cancer between June 2017 and June 2021. We considered patients older than 70 years as elderly patients, because several studies have shown that there is a statistically significant difference in surgical outcomes between gastric cancer patients older than 70 years and those younger than 70 years [13, 15, 16]. The inclusion criteria were as follows: patients ≥ 70 years old, gastric cancer was diagnosed by preoperative gastroscopy and pathological biopsy, patients underwent laparoscopic or open distal gastrectomy with D2 lymph node dissection, patients undergo curative resection, and the clinicopathological data were complete. Exclusion criteria: patients < 70 years old, other pathological types, distant metastasis, and lost to follow-up. According to the inclusion and exclusion criteria, a total of 303 patients’ clinical data were collected (laparoscopic = 178, open = 125). After 1:1 propensity score matching, 124 cases in each group were successfully included for analysis. Laparoscopic and open procedures were performed by the same trained team. This study was approved by the Medical Ethics Committee of Northern Jiangsu People’s Hospital. All patients and their families signed the informed consent before surgery.

### Surgical technique

#### Laparoscopic group

The laparoscopic operation holes are located at the lower edge of the umbilicus (observation hole), 1 cm below the intersection of the left axillary front line and rib edge (main operation hole), the horizontal intersection of the left clavicle midline and the umbilicus (auxiliary hole), 1 cm below the intersection of the right axillary front line and rib edge (auxiliary operation hole), and the horizontal intersection of the right clavicle midline and the umbilicus (auxiliary operation hole). The gastrocolic ligament was separated in the avascular area at the upper edge of the transverse colon, and the greater omentum was mobilized left to the splenic flexure of the colon and right to the hepatic region of the colon. Ligating and cutting off the left gastroepiploic artery and vein and then separating the 4sb group lymph nodes. The right gastroepiploic vessel was cut off, and the 6 group lymph nodes were dissected. The gastroduodenal artery, proper hepatic artery, and right gastric artery were separated and dissected along the root of the right gastroepiploic artery. The right gastric artery was ligated, and the 12a and 5 groups of lymph nodes were dissected. The left gastric artery was dissected and ligated, followed by dissection of the 7 and 8 groups of lymph nodes. The splenic artery was dissected posterior to the pancreas, and the 9 and 11p groups of lymph nodes were dissected. The lesser omentum was dissociated along the lower margin of the liver, and the 1 and 3 groups of lymph nodes were dissected. The duodenal bulb was dissociated 2 cm below the pylorus, and the duodenum was amputated with a linear cutting stapler. A 5–6 cm incision was made in the middle of the upper abdomen, and the stomach and omentum were extruded from the abdominal cavity, and then most of the distal stomach and tumors were removed. Finally, Billroth-I, Billroth-II, and Roux-en-Y were used for digestive tract reconstruction.

#### Open group

The surgical incision was made from the xiphoid process to 2 cm below the umbilicus. The gastrocolic ligament was separated in the avascular area at the upper edge of the transverse colon, and the greater omentum was mobilized left to the splenic flexure of the colon and right to the hepatic region of the colon. The left and right gastroepiploic vessels were ligated and cut off, and then, the 4 and 6 groups of lymph nodes were dissected. The gastric curvature was dissected, the right gastric artery was ligated, and then, the 5 group lymph nodes were dissected. The duodenal bulb was dissociated 2 cm below the pylorus, and the duodenum was amputated with a linear cutting stapler. The lesser omentum was dissociated along the lower margin of the liver, and the 1, 3, and 12a groups lymph nodes were dissected. The left gastric artery, common hepatic artery, and splenic artery were dissected, and the 7, 8, 9, and 11p groups of lymph nodes were dissected. Finally, most of the distal stomach and the tumor were removed, and the digestive tract was reconstructed with Billroth-I, Billroth-II, and Roux-en-Y.

### Data collection

Clinical data collected included patient demographics and baseline characteristics: age, gender, body mass index (BMI), American Society of Anesthesiologists (ASA), hypertension, diabetes mellitus, cardiovascular, pulmonary, hepatic, history of abdominal surgery, tumor size, and tumor pathological staging. Outcomes of surgical results and short-term outcomes: reconstruction, retrieved lymph node number, operation time, estimated blood loss, intraoperative transfusion, pain scale, time to first flatus, time to first liquid diet, postoperative hemoglobin, postoperative leukocytes, and hospital stays. Postoperative complications included wound infection, intra-abdominal abscess, intra-abdominal bleeding, intestinal obstruction, anastomotic bleeding, anastomotic stenosis, anastomotic leakage, pulmonary, urinary tract infection, cardiac complications, reoperation, and readmission. Routine follow-up was recommended for each patient: every 3 to 6 months for the first 2 years, every 6 to 12 months for the third to fifth years, and annually thereafter.

### Statistical analysis

SPSS 26.0 statistical software (IBM Corporation, Armonk, NY) was used to analyze the data. Continuous variables conforming to normal distribution were expressed as mean ± SD, and a comparison between the two groups was analyzed using the *t* test. Categorical variables were expressed as numbers (%), and a comparison between the two groups was analyzed using the chi-square test or Fisher’s exact test. Recurrence-free survival (RFS) was defined as the time from surgery to the time of recurrence or death from any cause. Overall survival (OS) was defined as the period between surgery and all-cause death. Patients who were lost to follow-up were censored. Kaplan–Meier curve and log-rank test were used for the evaluation of OS and DFS. The variables used to calculate the propensity score matching were as follows: sex, age, BMI, ASA, history of diabetes, and history of hypertension. The matching algorithm was a 1:1 nearest neighbor matching method with a caliper of 0.2 standard deviations. *P* < 0.05 was considered statistically significant.

## Results

**Fig. 1:**
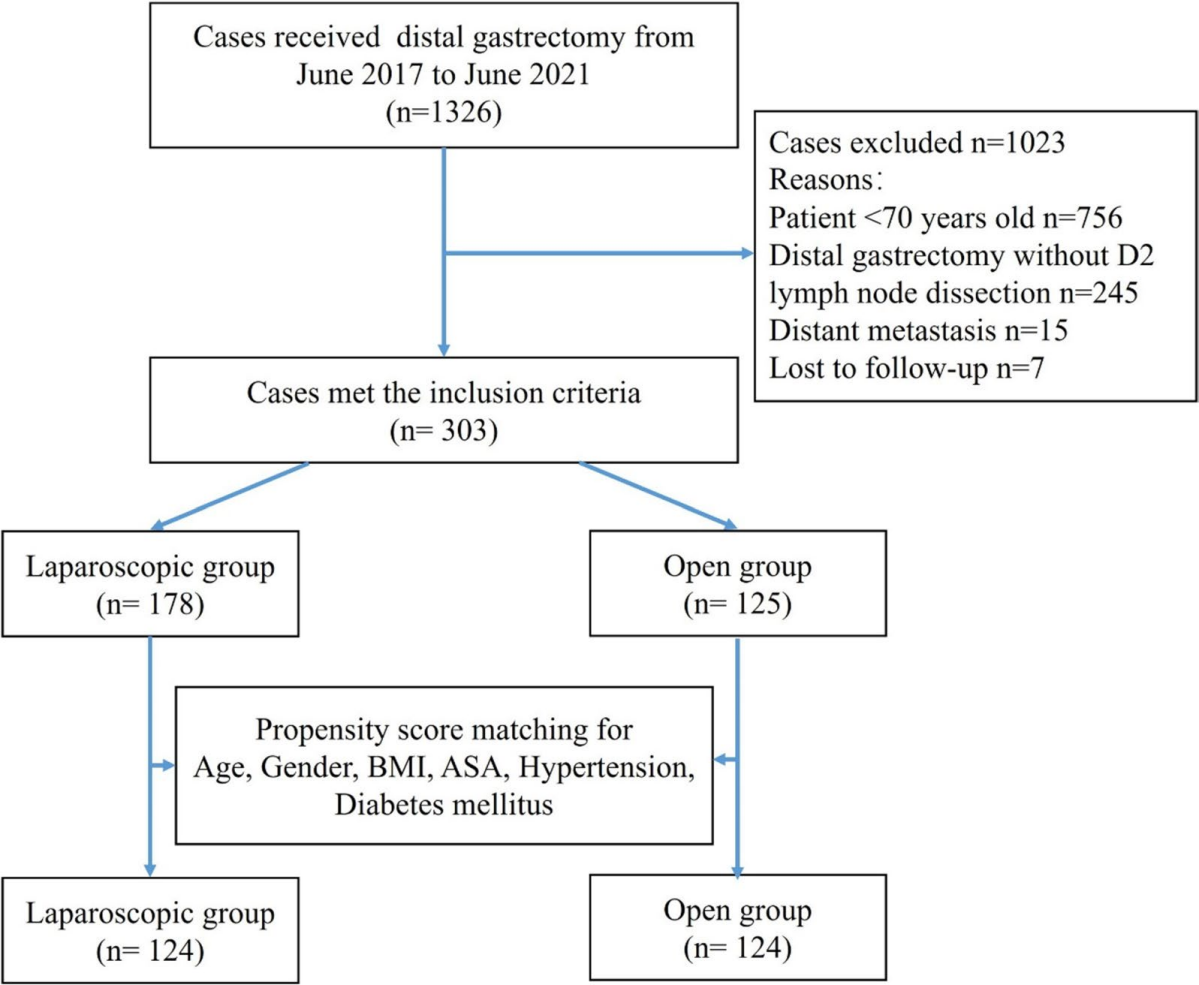
Flowchart of the inclusion criteria for the study: BMI body mass index. ASA American Society of Anesthesiologists

A total of 303 elderly patients who underwent laparoscopic or open distal gastrectomy for advanced gastric cancer between June 2017 and June 2021 met the inclusion criteria. Among them, 178 patients who underwent laparoscopic surgery were selected as the laparoscopic group, and 125 patients who received open surgery were classified as the open group. The 124 laparoscopic surgery patients and 124 open surgery patients were matched with propensity scores to reduce the impact of confounding factors in the analysis (Fig. 1). Before propensity score matching, the average BMI of the laparoscopic group was considerably higher than that of the open group (*P* = 0.015). After matching, no significant difference was found in any of the patients’ characteristics (Table 1).

**Table 1:** Patient demographics and baseline characteristics

Variable	Unadjusted		*P* value	Propensity score matched		*P* value
	Laparoscopic	Open		Laparoscopic	Open	
	(*N* = 178)	(*N* = 125)		(*N* = 124)	(*N* = 124)	
Age (years), mean (SD)	75.26 (2.82)	74.79 (2.38)	0.121	74.91 (2.68)	74.82 (2.37)	0.98
Gender *N* (%)						
Female	78 (43.8%)	56 (44.8%)		59 (47.6%)	56 (45.2%)	0.702
Male	100 (56.2%)	69 (55.2%)	0.866	65 (52.4%)	68 (54.8%)	
BMI (kg/m^2^), mean (SD)	24.23 (2.87)	23.48 (2.47)	0.015	23.57 (2.70)	23.51 (2.45)	0.848
ASA class, *N* (%)						
I/II	113 (63.5%)	82 (65.6%)		82 (66.1%)	82 (66.1%)	> 0.999
III/IV	65 (36.5%)	43 (34.4%)	0.705	42 (33.9%)	42 (33.9%)	
Diabetes, *N* (%)	36 (20.2%)	25 (20.0%)	0.962	24 (19.4%)	25 (20.2%)	0.873
Hypertension, *N* (%)	63 (35.4%)	43 (34.4%)	0.858	44 (35.5%)	43 (34.7%)	0.894
Cardiovascular, *N* (%)	18 (10.1%)	9 (7.2%)	0.381	12 (9.7%)	9 (7.3%)	0.494
Pulmonary, *N* (%)	25 (14.0%)	16 (12.8%)	0.755	18 (14.5%)	15 (12.1%)	0.575
Hepatic, *N* (%)	17 (9.6%)	11 (8.8%)	0.824	12 (9.7%)	11 (8.9%)	0.827
History of abdominal surgery, *N* (%)	17 (9.6%)	9 (7.2%)	0.472	11 (8.9%)	9 (7.3%)	0.641
Tumor size (cm), mean (SD)	4.20 (1.26)	4.43 (1.60)	0.171	4.19 (1.25)	4.43 (1.60)	0.187
pT-stage						
T1–T2	106 (59.6%)	78 (62.4%)		82 (66.1%)	77 (62.1%)	0.508
T3–T4	72 (40.4%)	47 (37.6%)	0.617	42 (33.9%)	47 (37.9%)	
pN-stage						
N0–N1	137 (77.0%)	96 (76.8%)		96 (77.4%)	95 (76.6%)	0.88
N2–N3	41 (23.0%)	29 (23.2%)	0.973	28 (22.6%)	29 (23.4%)	
p-stage						
I–II	109 (61.2%)	83 (66.4%)		88 (71.0%)	82 (66.1%)	0.412
III	69 (38.8%)	42 (33.6%)	0.358	36 (51.8%)	42 (33.9%)	
Histologic grade, *N* (%)						
Moderately differentiated	73 (41.0%)	47 (37.6%)		45 (36.3%)	46 (37.1%)	0.895
Poorly differentiated	105 (59.0%)	78 (62.4%)		79 (63.7%)	78 (62.9%)	

The characteristics of tumor pathological staging, including pT-stage (*P* = 0.5508), pN-stage (*P* = 0.88), p-stage (*P* = 0.4412), and histologic grade (*P* = 0.895), were similar between the two groups (Table 1). The surgical results and short-term outcomes of the two groups are shown in Table 2. In the outcome of operation time, laparoscopic surgery is significantly inferior to open surgery for distal gastric cancer in elderly patients (*P* < 0.001). Compared to the open group, the level of operative blood loss was noticeably lower in the laparoscopic group (*P* < 0.001). In the laparoscopic group, the pain scale on the first postoperative day was markedly lighter than the open group (*P* = 0.002). The laparoscopic group indicated significant superiorities in time to the first stool (*P* = 0.004) and time to first diet (*P* = 0.005) than the open group. Moreover, there were no significant differences between the two groups in type of reconstruction (*P* = 0.673), retrieved LN number (*P* = 0.113), intraoperative transfusion (*P* = 0.527), postoperative hemoglobin (*P* = 0.226), and postoperative leukocytes (*P* = 0.103).

**Table 2:** Surgical results and short-term outcomes

Variable	Unadjusted		*P* value	Propensity score matched		*P* value
	Laparoscopic	Open		Laparoscopic	Open	
	(*N* = 178)	(*N* = 125)		(*N* = 124)	(*N* = 124)	
Reconstruction, *N* (%)						
Billroth-I	44 (23.4%)	25 (20.0%)	0.257	27 (21.8%)	24 (19.4%)	0.673
Billroth-II	67 (35.6%)	37 (29.6%)		41 (33.1%)	37 (29.8%)	
Roux-en-Y	77 (41.0%)	63 (50.4%)		56 (45.2%)	63 (50.8%)	
Retrieved LN number, mean (SD)	26.20 (6.22)	27.56 (5.30)	0.042	26.38 (6.30)	27.56 (5.32)	0.113
Operation time (min)	194.19 (16.74)	187.72 (14.83)	0.001	195.48 (16.44)	187.78 (14.88)	< 0.001
Estimated blood loss (mL)	87.72 (10.25)	97.96 (14.54)	< 0.001	87.96 (10.19)	97.90 (14.58)	< 0.001
Intraoperative transfusion, *N* (%)						
No	157 (88.2%)	113 (90.4%)	0.545	110 (88.7%)	113 (91.1%)	0.527
Yes	21 (11.8%)	12 (9.6%)		14 (11.3%)	11 (8.9%)	
Pain scale, mean (SD)						
POD 1	4.98 (1.06)	5.35 (0.88)	0.002	4.98 (1.02)	5.35 (0.89)	0.002
POD 2	4.25 (1.10)	4.52 (1.05)	0.035	4.24 (1.01)	4.51 (1.05)	0.53
POD 3	2.90 (0.91)	3.07 (1.00)	0.132	2.83 (0.92)	3.06 (1.00)	0.056
Time to first flatus (days), mean (SD)	3.62 (0.90)	3.94 (1.00)	0.003	3.61 (0.84)	3.95 (1.00)	0.004
Time to first liquid diet (days), mean (SD)	4.15 (1.07)	4.56 (1.02)	0.001	4.19 (1.10)	4.56 (1.02)	0.005
Postoperative hemoglobin (g/L), mean (SD)	121.08 (18.30)	118.54 (19.92)	0.252	121.55 (18.67)	118.56 (20.00)	0.226
Postoperative leukocytes (*10^9/L), mean (SD)						
(*10^9/L), mean (SD)	8.68 (3.24)	9.26 (3.07)	0.119	8.59 (3.12)	9.23 (3.07)	0.103
Hospital stays (days), mean (SD)	9.01 (2.21)	10.16 (2.72)	< 0.001	9.00 (2.15)	10.15 (2.73)	< 0.001

The postoperative complications of the two groups were presented in Table 3. In the outcome of total complications, the laparoscopic group indicated significant superiorities than the open group (*P* = 0.011). Compared to the open group, the laparoscopic group possessed fewer complications containing wound infection (*P* = 0.281), intra-abdominal bleeding (*P* = 0.582), intestinal obstruction (*P* = 0.175), anastomotic stenosis (*P* > 0.999), anastomotic leakage (*P* = 0.519), pulmonary (*P* = 0.121), urinary tract infection (*P* = 0.641), cardiac complications (*P* > 0.999), reoperation (*P* = 0.719), and readmission (*P* > 0.999), but no significant difference was found between the two groups.

**Table 3:** Postoperative complications

Variable	Unadjusted		*P* value	Propensity score matched		*P* value
	Laparoscopic	Open		Laparoscopic	Open	
	(*N* = 178)	(*N* = 125)		(*N* = 124)	(*N* = 124)	
Wound infection, *N* (%)	4 (2.2%)	6 (4.8%)	0.369	2 (1.6%)	6 (4.8%)	0.281
Intra-abdominal abscess, *N* (%)	5 (2.8%)	2 (1.6%)	0.758	2 (1.6%)	2 (1.6%)	> 0.999
Intra-abdominal bleeding, *N* (%)	12 (6.7%)	7 (5.6%)	0.687	8 (6.5%)	6 (4.8%)	0.582
Intestinal obstruction, *N* (%)	5 (2.8%)	4 (3.2%)	> 0.999	1 (0.8%)	4 (3.2%)	0.175
Anastomotic bleeding, *N* (%)	7 (3.9%)	5 (4.0%)	> 0.999	5 (4.0%)	5 (4.0%)	> 0.999
Anastomotic stenosis, *N* (%)	4 (2.2%)	3 (2.4%)	> 0.999	2 (1.6%)	3 (2.4%)	> 0.999
Anastomotic leakage,* N* (%)	6 (3.4%)	6 (4.8%)	0.742	4 (3.2%)	6 (4.8%)	0.519
Pulmonary, *N* (%)	8 (4.5%)	11 (8.8%)	0.128	5 (4.0%)	11 (8.9%)	0.121
Urinary tract infection, *N* (%)	15 (8.4%)	12 (9.6%)	0.724	9 (7.3%)	11 (8.9%)	0.641
Cardiac complications, *N* (%)	4 (2.2%)	3 (2.4%)	> 0.999	2 (1.6%)	3 (2.4%)	> 0.999
Reoperation, *N* (%)	7 (3.9%)	5 (4.0%)	> 0.999	3 (2.4%)	5 (4.0%)	0.719
Readmission, *N* (%)	5 (2.8%)	4 (3.2%)	> 0.999	3 (2.4%)	4 (3.2%)	> 0.999
Total complications, *N* (%)	82 (46.1%)	68 (54.4%)	0.153	46 (37.1%)	66 (53.2%)	0.011

**Fig. 2:**
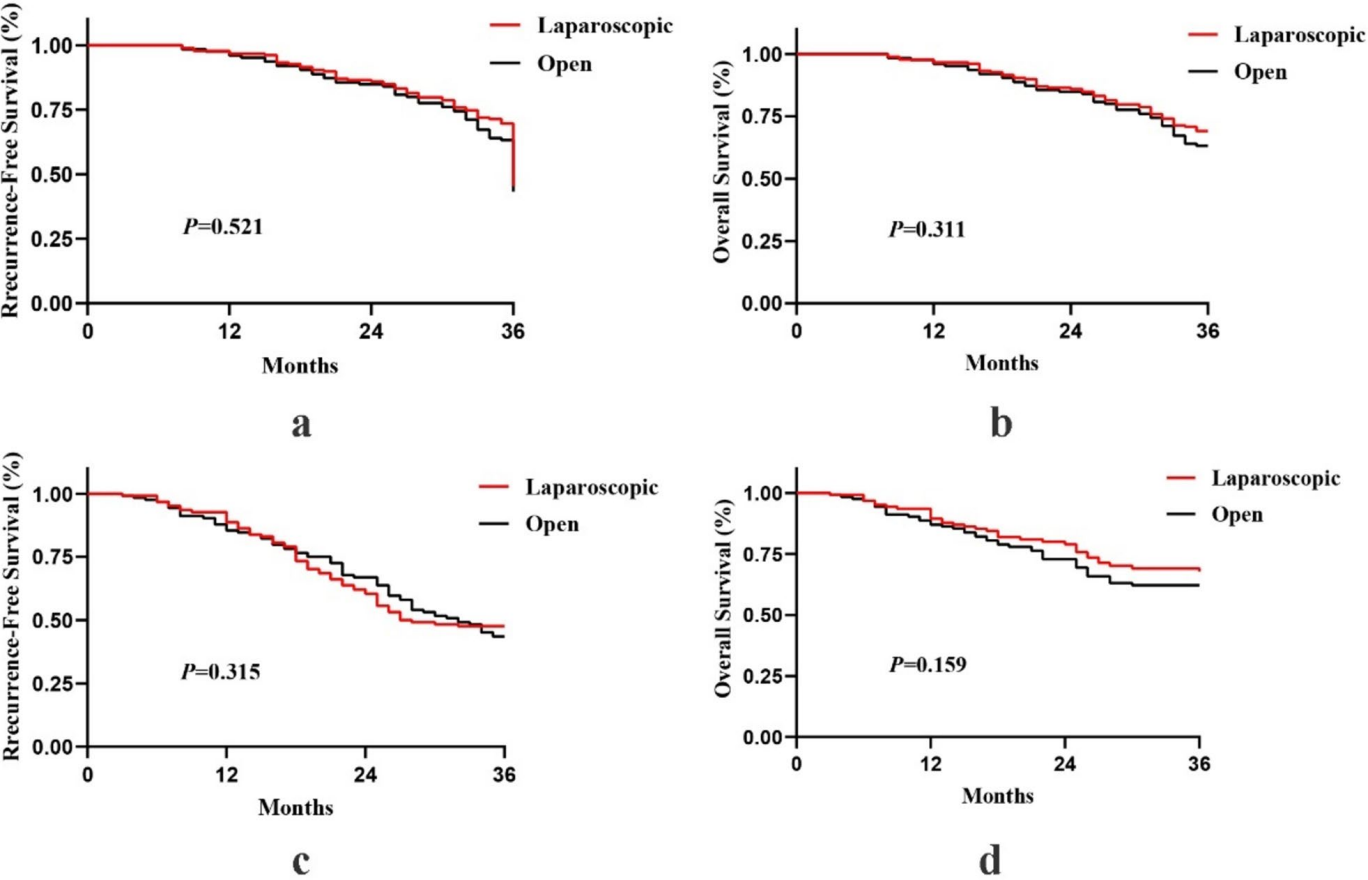
Kaplan–Meier analysis of 3-year recurrence-free survival (RFS) and overall survival (OS) for laparoscopic versus open groups: **a** RFS before propensity score matching. **b** OS before propensity score matching. **c** RFS after propensity score matching. d OS after propensity score matching

We used the Kaplan–Meier survival analysis to evaluate 3-year RFS and OS for laparoscopic versus open groups (Fig. 2). There was no significant difference in the RFS (*P* = 0.521) rates and OS (*P* = 0.311) rates between the two groups. Moreover, we found no noticeable difference in the 3-year RFS (*P* = 0.315) rates and OS (*P* = 0.159) rates between the two groups after the propensity score matched.

## Discussion

Nowadays, laparoscopic distal gastrectomy with D2 lymph node dissection has been widely used in clinical practice [1]. However, the role of laparoscopic distal gastrectomy in elderly patients is rarely reported. Our study aimed to explore the short-term and mid-term efficacy of laparoscopic and open surgery in radical distal gastrectomy. For patients with advanced gastric cancer, the level of blood loss was obviously higher in the open group than that in the laparoscopic group. We found that the number of lymph node dissection and intraoperative blood transfusions were similar between the laparoscopic and open groups. However, the operation time was distinctly longer in the laparoscopic group. Notably, the level of pain scale on the first postoperative day was markedly lighter than the open group, although no significant differences were shown on the second and third postoperative days. As expected, the laparoscopic group had apparent superiorities in time to first flatus, time to first liquid diet, and hospital stays. Moreover, the incidence of total complications was noticeably lower in the laparoscopic group than the open group. However, in these specific complications, there was no significant difference between the two groups. Three-year RFS and OS were similar between the two groups.

In laparoscopic gastric cancer surgery, the establishment of intraoperative pneumoperitoneum has a certain impact on the patient’s respiratory and circulatory system [17]. In addition, the deterioration of cardiopulmonary function in elderly patients may lead to a decrease in surgical tolerance, which possibly has an impact on the safety of surgery [18]. However, our study found no significant difference in the number of lymph node dissection, intraoperative blood transfusion, and postoperative complications between the two groups. Moreover, the laparoscopic group showed prominent advantages over the open group in terms of intraoperative blood loss, postoperative gastrointestinal function recovery, and length of hospital stay, which indicated that laparoscopic distal gastrectomy is safe and effective in elderly patients. Yen et al. [15] reported that laparoscopic distal gastrectomy for gastric cancer had obvious advantages, including faster recovery and fewer complications compared with open surgery in a retrospective study. The above results manifest that laparoscopic distal gastrectomy is more suitable for elderly patients.

Similar to previous studies, the operative time in the laparoscopic group was significantly longer than that in the open group. Hakkenbrak et al. [19] conducted a meta-analysis of 22 randomized clinical trials and found that laparoscopic distal gastric cancer surgery required longer operation time than open surgery and suggested that this may be related to the learning curve effect of laparoscopic surgery. Kitano et al. [20] found that the operation time of laparoscopy-assisted total gastrectomy was significantly longer than open surgery in a multicenter study of 1924 patients with gastric cancer. Kitano suggested that the reason for the difference in operation time was related to tumor size and T stage of the tumor. Shi et al. [7] considered that establishing pneumoperitoneum, replacing instruments, cleaning cameras, and performing minor incisions during laparoscopic gastric cancer surgery increased overall operation time. In addition, we suspect that the operation time is greatly related to the level of the surgeon’s laparoscopic technique, knowledge of the anatomy of the stomach and surrounding tissues, and surgical experience. The surgeon’s unclear anatomical relationship between the stomach and surrounding tissues may cause unnecessary damage to surrounding blood vessels and organs, which may require more time to deal with intraoperative complications such as vascular bleeding and organ damage [21, 22]. However, the operation time of laparoscopic radical distal gastrectomy will be gradually shortened with the continuous development of laparoscopic technology, the improvement of the surgeon’s technical level, and the accumulation of surgical experience [23, 24].

This study showed that laparoscopic surgery was superior to open surgery in postoperative recovery of gastrointestinal function. Wang et al. [25] conducted a retrospective study on 1360 patients who underwent radical distal gastrectomy and found that the laparoscopic group was significantly superior to the open group in time to first flatus, time to first liquid diet, and hospital stays. Lou et al. [26] reported that laparoscopic surgery had significant advantages in postoperative recovery and hospital stay in a meta-analysis of 7643 gastric cancer patients. We think that the factors affecting the recovery of gastrointestinal function may include surgical resection range, digestive tract reconstruction method, and postoperative pain level [27]. In this study, there was no significant difference in the methods of digestive tract reconstruction between the two groups, but the pain level on the first postoperative day was significantly lower in the laparoscopic group than in the open group. The alleviation of postoperative pain after laparoscopic surgery is beneficial for patients to get out of bed as soon as possible, which plays a vital role in the recovery of gastrointestinal function. In addition, earlier ambulation can also help reduce the risk of thrombosis and pulmonary infection caused by long-term bed rest, so as to promote postoperative recovery of patients.

The present study has certain limitations. Firstly, this was a retrospective study with a small number of cases, resulting in an inevitable error between the final and actual results. In addition, the follow-up period of the patients in this study was three years, lacking longer-term follow-up results, which has certain limitations in evaluating the safety and effectiveness of the two surgical methods. Furthermore, patients who received neoadjuvant therapy were not included in the study. Therefore, the efficacy of laparoscopic distal gastrectomy after neoadjuvant therapy is still unclear and needs to be confirmed by further studies. This study was based on a dataset of Chinese hospitals. However, the volume of distal gastric cancer surgery and the proficiency of surgeons vary from country to country. Therefore, the safety and efficacy of laparoscopy in distal gastric cancer radical surgery still need to be demonstrated through a larger number of cases and multicenter randomized clinical trials.

## Conclusion

Our results suggest that laparoscopic distal gastrectomy has the advantages of less intraoperative bleeding, lower postoperative pain scales, faster recovery of gastrointestinal function, and shorter hospital stays in elderly patients with gastric cancer compared to open surgery. The laparoscopic and open surgery groups showed similar postoperative complications and 3-year follow-up results, indicating that laparoscopic distal gastrectomy is safe and effective for treating elderly patients with gastric cancer.

## Data Availability

The data that support the findings of this study are available from the corresponding author upon request.
